# Discovery of a novel IL-15 based protein with improved developability and efficacy for cancer immunotherapy

**DOI:** 10.1038/s41598-018-25987-4

**Published:** 2018-05-16

**Authors:** Qiyue Hu, Xin Ye, Xiangdong Qu, Dongbing Cui, Lei Zhang, Zhibin Xu, Hong Wan, Lianshan Zhang, Weikang Tao

**Affiliations:** Shanghai Hengrui Pharmaceutical Co. Ltd., 279 Wenjing Road, Shanghai, 200245 China

## Abstract

Interleukin-15 (IL-15) can promote both innate and adaptive immune reactions by stimulating CD8^+^/CD4^+^ T cells and natural killer cells (NK) while showing no effect in activating T-regulatory (Treg) cells or inducing activation-associated death among effector T cells and NK cells. Thus, IL-15 is considered as one of the most promising molecules for antitumor immune therapy. To improve the drug-like properties of natural IL-15, we create an IL-15-based molecule, named P22339, with the following characteristics: 1) building a complex of IL-15 and the Sushi domain of IL-15 receptor α chain to enhance the agonist activity of IL-15 via transpresentation; 2) through a rational structure-based design, creating a disulfide bond linking the IL-15/Sushi domain complex with an IgG1 Fc to augment its half-life. P22339 demonstrates excellent developability, pharmacokinetic and pharmacodynamic properties as well as antitumor efficacy in both *in vitro* assessments and *in vivo* studies. It significantly suppresses tumor growth and metastasis in rodent models, and activates T effector cells and NK cells in cynomolgus monkey. Overall, these data suggest that P22339 has a great potential for cancer immunotherapy.

## Introduction

Interleukin 15 (IL-15) is a cytokine of about 12–14 KD discovered by Grabstein *et al*. in 1994^1^. It is a growth factor for T cells and NK cells, and plays an important role in the development, proliferation and activation of these immune cells. IL-15 has been identified by the American National Cancer Institute as one of the most promising immunotherapy targets for cancer^2^. A related cytokine IL-2 has been approved by the US Food and Drug Administration for the treatment of metastatic renal cell carcinoma and malignant melanoma. However, its application is limited due to its stimulatory effects on Treg cells and its induction of the Activation-Induced Cell Death (AICD) among T effect cells and NK cells. Both processes can limit the memory T cell response and induce T cell tolerance. Both IL-15 and IL-2 bind to the IL-15 receptor (IL-15R) β and γ_c_. Unlike IL-2, IL-15 neither interacts with Treg cells nor induces AICD^3,4^, therefore avoiding the liabilities of IL-2.

IL-15 binds to the IL-15R which consists of α, β, and γ_c_ chains. IL-15Rβ (also known as IL-2Rβ or CD122) and IL-15Rγ_c_ (also known as CD132) can bind to both IL-15 and IL-2 with intermediate affinity. IL-15Rα is widely expressed and only binds IL-15 with high affinity. IL-15Rα contains a Sushi domain (1–65 amino acids), which is responsible for interacting with IL-15, and is essential for mediating the biological function of IL-15^5^.

Although IL-15 has a great potential for therapeutic use^6^, natural IL-15 has obvious druggability issues, namely a low biological potency, a short half-life^7^, and an inferior developability^8,9^. Therefore, to develop an IL-15 based therapeutic agent, it is essential to enhance its potency, extend its half-life and improve its developability. A number of studies indicate that the complex formed by IL-15 and the Sushi domain of soluble IL-15Rα is significantly stronger than IL-15 alone in stimulating the proliferation of memory CD8^+^ T lymphocytes and NK cells, as well as maintaining the viability of memory CD8^+^ T cell through the trans-presentation mechanism^7,10–13^. That suggests creating an IL-15/Sushi domain fusion protein can significantly increase potency. It has been reported that a covalent linkage formed between the two binding proteins by disulfide bond can make significant contribution to the stability of the complex primarily due to the decrease in the entropy of the unfolded protein^14^. Rational design of disulfide bond has been widely used to improve the protein stability, facilitate the research of protein function, and generate novel therapeutic proteins^15–19^. In this study, we employed a two-step computational modeling approach to engineer a new disulfide bond between IL-15 and the Sushi domain of IL-15Rα based on the published co-crystal structure (^20^; PDB ID: 2Z3Q). In step 1, we selected the potential mutation sites to pair disulfide bonds. In step 2, we evaluated the energetic effects on the protein stability for the selected residues found in step 1. A subset of the mutation combinations were selected and compared by protein expression, *in vitro* functional assay and stability. L52C of IL-15 and S40C of IL-15Rα were chosen as the final mutation combination based on the modeling result. There are multiple ways to improve the half-life of a protein molecule^21^. We chose to fuse the Sushi domain with the Fragment Crystallizable region (Fc) of a human IgG1, and generated a Fc-fusion molecule for improving the PK properties. The resulted molecule, P22339, was then evaluated with the biological activities, developability, pharmacokinetic and pharmacodynamic properties. The *in vivo* studies demonstrated the antitumor immunity of P22339 through the induced proliferations of NK cells, CD8^+^ and CD4^+^ T cells which resulted in tumor inhibition.

## Results

### Design of IL-15 analogs for improving biological activities and developability

**Figure 1:**
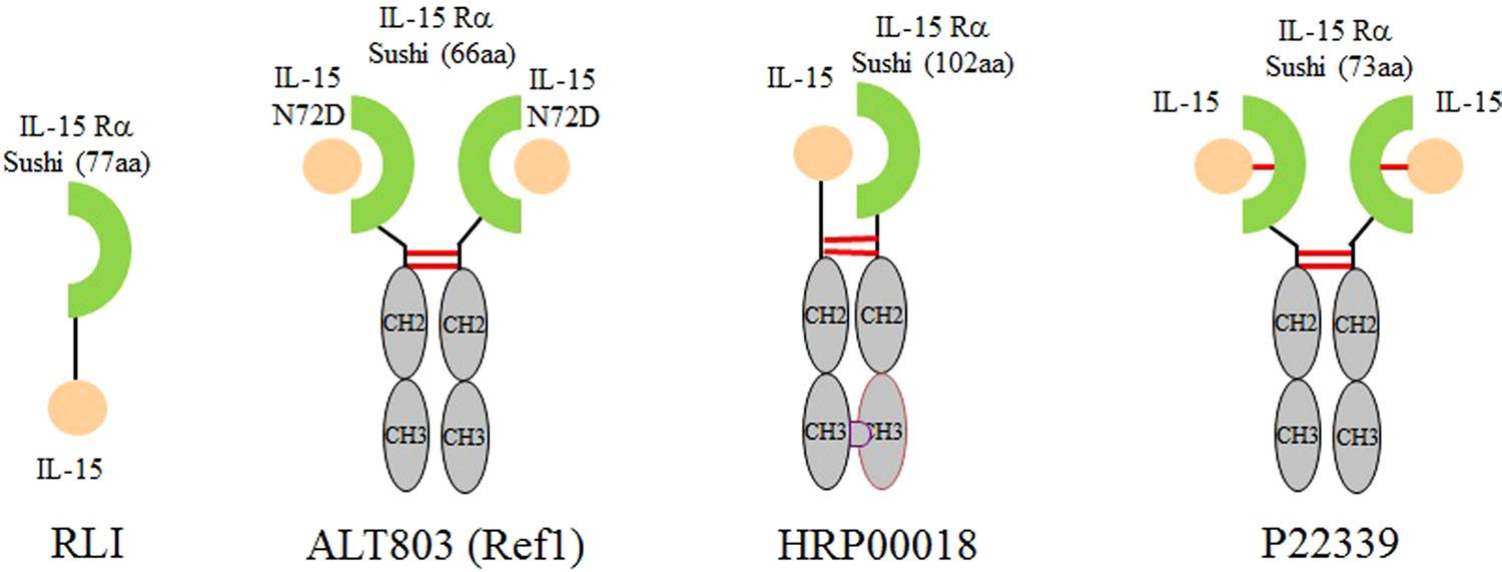
Schematic Drawings of selected IL-15 and Receptor α complex molecules. IL-15 is displayed as orange ball (mutations are indicated in the picture). IL-15 receptor α motif is represented by green half circle (The number of the amino acids is shown in parentheses). Fc domain is illustrated with gray oval. Intermolecular disulfide bond is represented with solid red line.

There have been many companies or institutions engaging in researches related to IL-15 immunotherapy^22–27^. Some of the representative IL-15 and Receptor α complex molecules are shown in Fig. 1. In order to mitigate those liabilities of IL-15 mentioned above, we designed our molecules from three perspectives: (1) Form complex with IL-15Rα to take advantage of the activity boosting effect; (2) Increase molecular weight to pass 50 KD with Fc fusion to reduce the renal filtration and degradation^21^; (3) Introduce intermolecular disulfide bond between IL-15 and its receptor α to further increase the molecular stability and bioactivity as well as improve the developability of the molecule.

#### Design and Selection of the disulfide bond pairs

We started from the crystal complex structure of human IL-15 and the receptor α, β, γ (^28^; PDB ID: 4GS7). It could be seen that the three receptors bind to IL-15 in three different orientations respectively (see Supplementary Fig. S1). Using 6Å as the cutoff, the residues located on the interface of IL-15 and receptor α were identified (see Supplementary Table S1). We hypothesized that we could modify the residues presented on the interface between IL-15 and receptor α while maintaining the binding of IL-15 to receptors β and γ.

**Figure 2:**
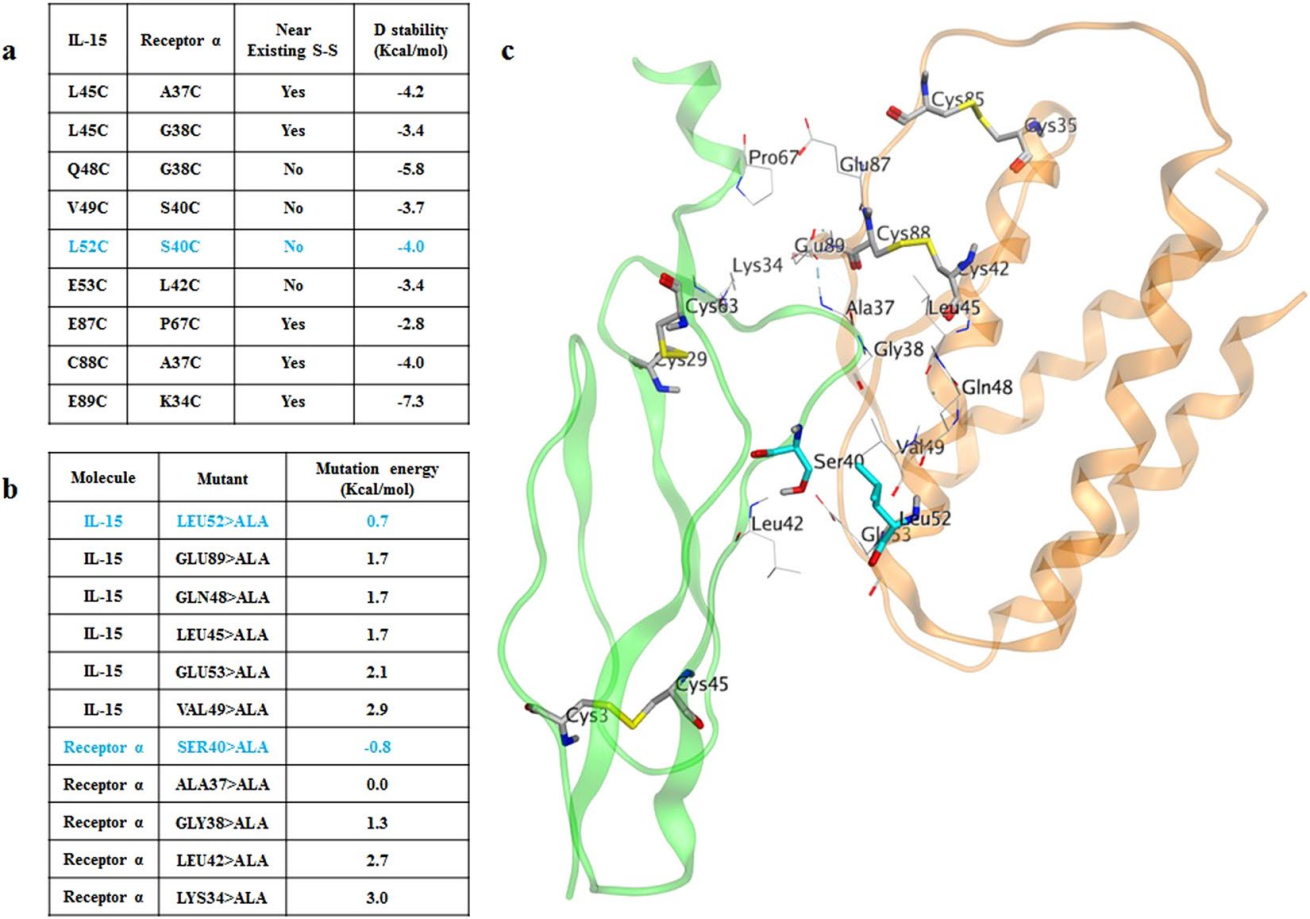
Structure-based inter-molecular disulfide bond design. (**a**) Selected disulfide bond pairs from Disulfide Scan. (**b**) Result from the *In silico* Alanine Scanning. The residues for the preferred amino acid pair are indicated in blue. (**c**) IL-15 is colored in orange and the receptor α is colored in green. The intra-molecular disulfide bonds within each molecule are rendered in stick mode. The selected mutation pair to form inter-molecular disulfide bond, S40C on Receptor α and L52C on IL-15, are highlighted in stick and colored in cyan.

Computational Disulfide Scan was performed at the interface between IL-15 and receptor α. Total 9 pairs of Cysteine mutations were selected (Fig. 2a). In order to further evaluate the stability of the mutations, *in silico* alanine scan was carried on all of the residues from the previous Disulfide Scan step. The calculated mutation energy changes (Fig. 2b) showed that L52A on IL-15 and S40A on IL-15Rα minimally affect the structural stability. Therefore, L52 from IL-15 and S40 from the IL-15Rα were considered as the preferred candidates for mutation engineering to Cysteine as shown in Fig. 2c.

Based on the computational results, eight mutational pairs were selected for cell expression. The specific co-expression combinations were shown in Supplementary Table S2. There were two groups with Cysteine mutation indicated in the name, 1) combinations 1–9, IL-15-6His co-expressed with IL-15Rα-Fc fusion molecule, and 2) combinations 10–18, IL-15-Fc fusion molecule co-expressed with IL-15Rα. The cell supernatant obtained from the co-expression was subjected to western analysis. The pairing of Fc-fused IL-15Rα and IL-15-6His could result in the correct productions of the intended complex molecule. The combination 5, 6, 7 showed the best result in terms of the expression levels, with single product in the desired MW range (see Supplementary Fig. S2a). At the same time, the co-expressions of Fc-fused IL-15 and IL-15Rα were prone to result in mismatch, and reduced the amount of the correctly paired target products (see Supplementary Fig. S2b). The results were in good agreement with the computational prediction. Based on the results above and the yields in CHO cell system, combination #6, L52C-S40C, was named as P22339 and selected for further characterizations.

#### Developability evaluation of P22339

Through two chromatography steps, we purified P22339 to 99.77% yield based on SEC-HPLC analysis (see Supplementary Fig. S3). Notably, there was no obvious purity change after the sample was frozen-thawed repeatedly for more than four cycles from −80 °C to room temperature.

In Non-reducing SDS-PAGE, the purified P22339 was found to be a clear single band below 115 KDa, which confirmed structure and purity. In reducing SDS-PAGE, the product was found to contain four bands with one molecular weight of 40 kDa and other three between 10 KDa and 25 KDa (see Supplementary Fig. S4a). After a digestion with N-Glycosidase F (PNGase F), only two proteins, with molecular weights of ~37 kDa and 13 kDa, were detected (see Supplementary Fig. S4b). These molecular weights closely matched the calculated molecular weights of IL-15Rα-Sushi-Fc and IL-15. This suggested that these two proteins were glycosylated during mammalian cell production and the IL-15 was produced in two major glycosylated forms and one minor non-glycosylated form.

#### *In vitro* potency of P22339

**Figure 3:**
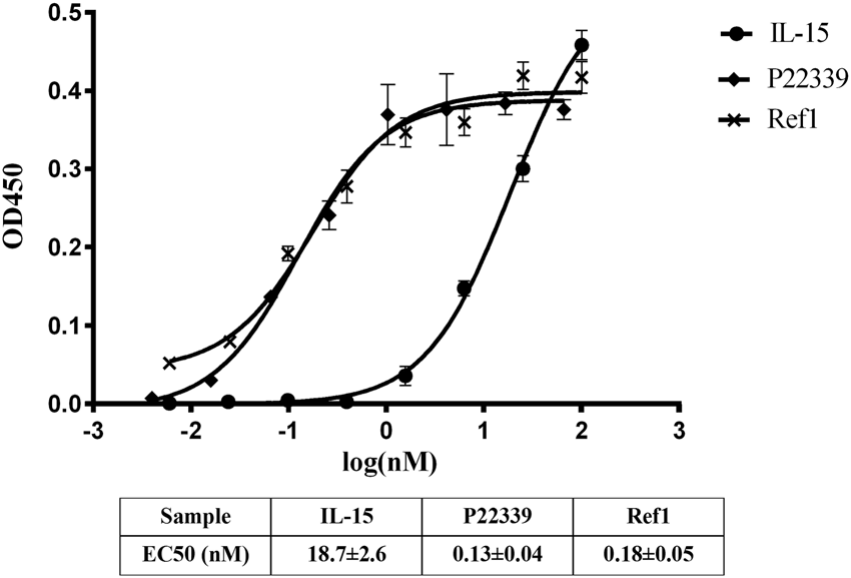
Mo7e cells were cultured with increasing concentrations of human recombinant IL-15, Ref1 and P22339. Mo7e proliferation was evaluated by Cell Counting Kit-8(CCK-8). Points, mean of triplicate wells of the same experiment. Error bars, SD.

Using IL-15 and Ref1 as the positive controls, we tested the potency of the rationally designed P22339 to assess the stimulation effects on the cellular proliferation in the Mo7e cell line which expressed human IL-15R β and γ but not IL-15Rα. At saturation, all three molecules tested were able to induce similar level of maximum proliferation. Compared to IL-15, P22339 was able to significantly improve the half-maximal of the proliferation (EC50) for Mo7e cell line by 143 folds, while Ref1 was 104 folds (Fig. 3). PBS was added as the negative control, which showed the same level of effect as blank signal and did not stimulate the cell growth and proliferation.

#### *In vivo* potency of P22339

P22339 was further tested in two *in vivo* models to assess the inhibition of the metastasis and tumor growth in B16F10 mice lung metastasis and syngenic tumor models.

**Figure 4:**
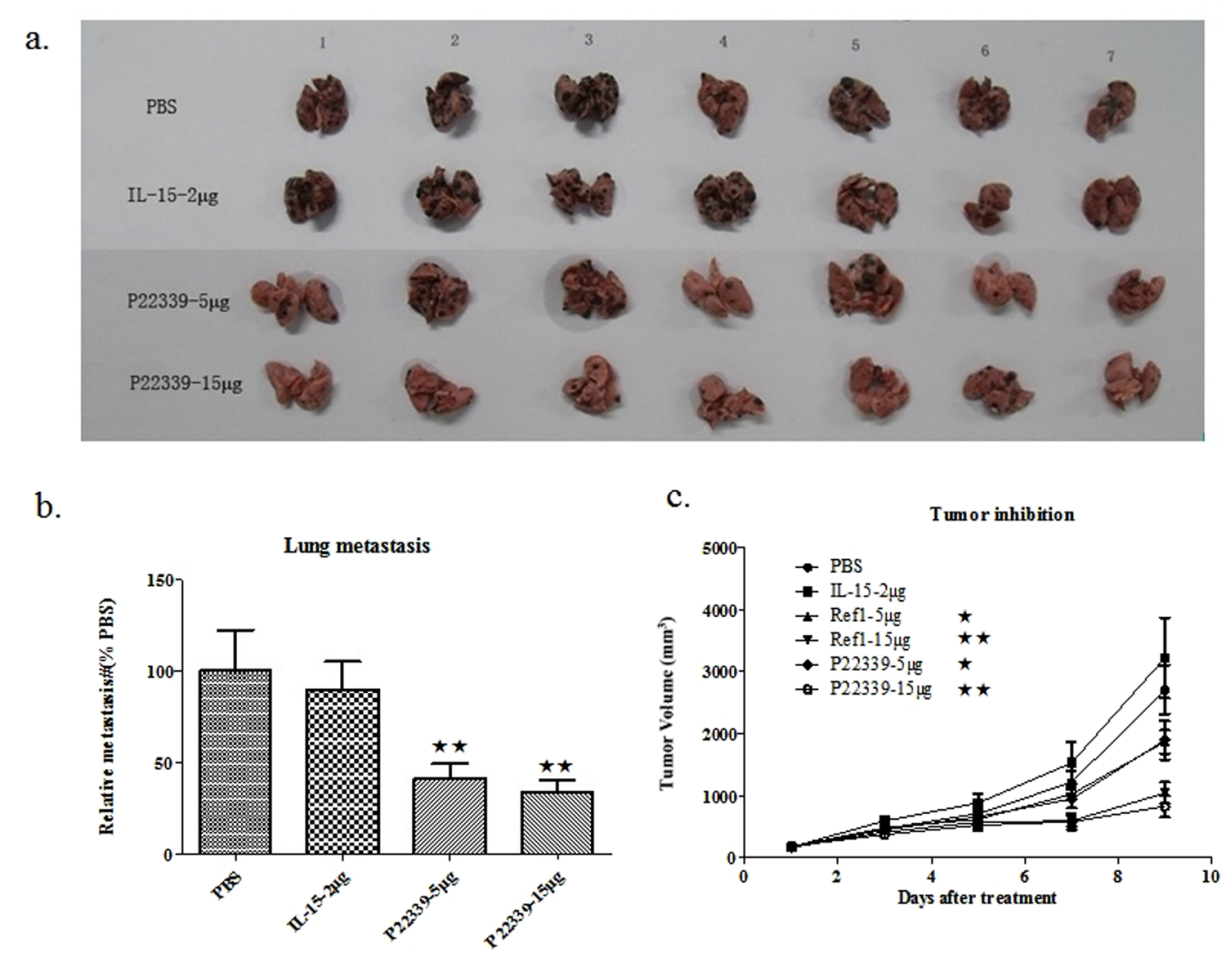
Therapeutic effects of systemically administered IL-15, P22339 and Ref1 in the mouse B16F10 models. (**a**) Four groups of mice (8 mice per group) were treated with IL-15, P22339, or PBS on days 1. On day 16, mice were sacrificed and tumors (black nodules) on the lungs of all animals were counted. (**b**) P22339 (2 μg and 15 μg) or IL-15 (2 μg) was administered IP on days 1, 2, and 10. Columns, mean of each group (n = 7); bars, SEM. ^★★^P < 0.01 versus control, Dunnett’s multiple comparison test. (**c**) Mice were injected subcutaneously in the right rib with B16F10 mouse melanoma cells, as described in the Methods. After 1 week (average tumor size 160 ± 40 mm^3^), mice were treated IP with: PBS (phosphate-buffered saline) control, IL-15 (2 μg), Ref1 (5 μg and 15 μg) or P22339 (5 μg and 15 μg) on day 1 and day 5. Each group consisted of 7 mice. Tumor volumes were measured three times a week. The mice were sacrificed 16 days after tumor injection. Bars, SEM. ^★^P < 0.05, ^★★^P < 0.01 versus control, Dunnett’s multiple comparison test.

C57BL/6 mice were inoculated with 1 × 10^5^ B16F10 melanoma cells on day 0 and treated intraperitoneally (IP) with IL-15 (2 μg/mouse) or P22339 (5 μg/mouse and 15 μg/mouse) at days 1, 2, and 10. At day 16, mice were sacrificed and the lung metastatic melanoma nodules visible as black spots (see Fig. 4a) were counted. In the metastasis model, compared to IL-15, P22339 was able to reduce the lung metastasis number without body weight reduction (Fig. 4a,b). The Lungs of mice in PBS group showed a large number of metastatic melanoma growing (73 ± 43). Lungs of IL-15 group showed marginal reduction in the number of melanoma lumps (65 ± 29), about 90% of that in PBS (phosphate-buffered saline) group. Lungs of 5 μg P22339 group showed a noticeable metastasis reduction of melanoma lumps (30 ± 16), about 41% of that in PBS group. Lungs of 15 μg P22339 group showed a similar metastasis reduction of melanoma lumps (24 ± 13), about 33% of that in PBS group. The relative lung metastasis number in PBS group was significantly higher than that in both P22339 dose groups. No apparent decrease in body weight was observed in each group during the administration, suggested that the administered dosages did not have significant toxicity.

In a separate syngeneic tumor model experiment, 42 C57 BL/6 mice divided in six groups were injected subcutaneously in the right rib with B16F10 mouse melanoma cancer cells. Mice were treated intraperitoneally with: PBS control, IL-15 (2 μg/mouse), Ref1 (5 μg/mouse and 15 μg/mouse) or P22339 (5 μg/mouse and 15 μg/mouse) on day 1 and day 5. The mice were sacrificed 16 days after tumor injection. Tumor volumes were measured three times a week. We observed dose dependent anti-tumor activity for P22339 at both 5 μg and 15 μg group, 30% and 73% respectively, while Ref1 also showed 31% and 60% at the same dose levels. In comparison, IL-15 was dosed at 2 μg/mouse without tumor shrinking effect (see Fig. 4c and Supplementary Table S3). No bodyweight lost was observed for any treatment groups.

**Figure 5:**
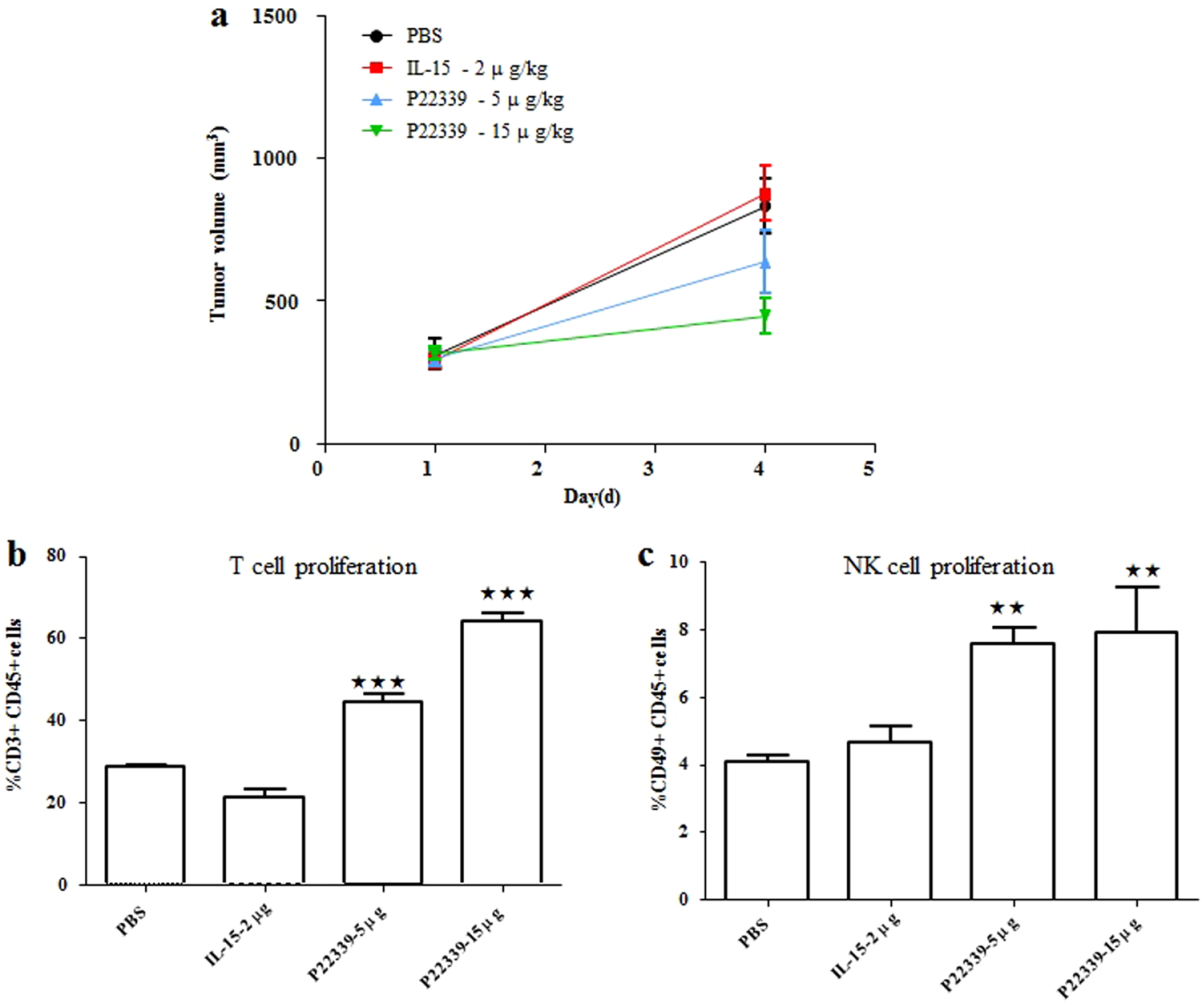
Tumor inhibition and immune cell proliferation result of IL-15 and P22339 in the 4 day treatment experiment using the B16F10 melanoma model in C57 mice. (**a**) Tumor volume trace after administering to female C57BL/6 mice bearing B16F10 tumors. Data points represent group mean, error bars represent standard error of the mean (SEM), n = 4. ^★★^Means p < 0.01. (**b**) The mean percentage of CD3^+^/CD45^+^ cells in all groups. (**c**) The mean percentage of CD49b^+^/CD45^+^ cells in all groups. Data points represent group mean, error bars represent standard error of the mean (SEM), n = 4. ^★★★^Means p < 0.001.

To further confirm the effect of antitumor efficacy was caused by the stimulation of T and NK cells, we carried out another *in vivo* experiment in the same tumor bearing B16F10 melanoma model in C57BL/6 mice. Mice were treated once on day 9 after tumor inoculation when the average tumor size reached approximately 300 mm^3^. The mean tumor size of the PBS control group reached 832 mm^3^ on day 4 after treatment. When compared to the PBS treatment, 2 μg IL-15 showed no tumor inhibition, while P22339 at 5 μg and 15 μg doses exhibited 30% and 46% antitumor activity respectively on day 4 after treatment. Tumor growth curves were shown in Fig. 5a. The effect of P22339 and IL-15 on the function of immune cell subpopulations were evaluated by flow cytometry technique. The percentages of CD3^+^/CD45^+^ cells were used to represent T cell proliferation. When compared to the 28.6% of the PBS group, IL-15 2 μg, P22339 5 μg and 15 μg groups showed 21.2%, 44.5% and 64.1%, respectively, as shown in Fig. 5b. The corresponding FACS plots were provided in Supplementary Fig. S5a. The percentages of CD49b^+^/CD45^+^ cells were used to represent the effect on NK cell proliferation^29^. When compared to the 4.1% of the PBS group, IL-15 2 μg, P22339 5 μg and P22339 15 μg groups showed 4.7%, 7.5% and 7.9%, separately, as shown in Fig. 5c. The corresponding FACS plots were provided in Supplementary Fig. S5b. The results showed that P22339 could significantly induce the proliferation of T and NK cells at 5 and 15 μg doses which caused tumor shrinkage in the B16F10 melanoma model in a dose dependent manner.

#### Pharmacokinetics in Rats

**Figure 6:**
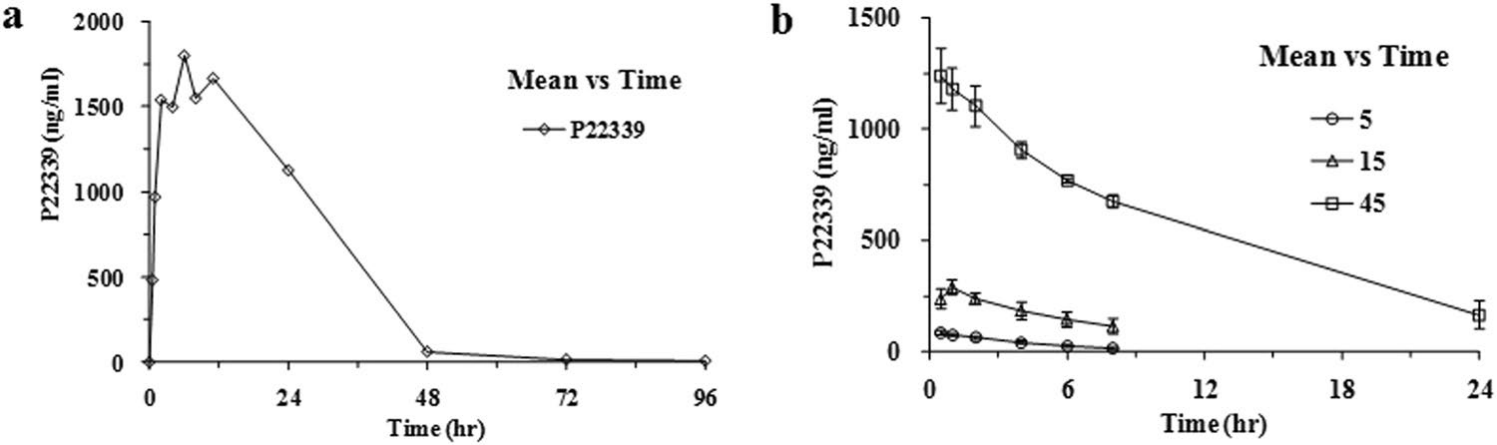
(**a**) Pharmacokinetics curve in rats. The plasma concentrations of P22339 were analyzed in blood samples obtained from SD rats (n = 2) before dosing and at 0.5 h, 1 h, 2 h, 4 h, 6 h, 8 h, 11 h, 24 h, 48 h, 72 h and 96 h, respectively, following a single IP injection with a dose of 188 μg/kg of P22339 after fasting overnight. (**b**) Pharmacokinetics curve in monkeys. The plasma concentrations of P22339 were analyzed in blood samples obtained from cynomolgus monkeys (n = 3/sex/group) before dosing and at 0 h, 0.5 h, 1 h, 2 h, 4 h, 8 h and 24 h, respectively, following a single IV injection dose of 5 µg/kg, 15 µg/kg and 45 µg/kg P22339. The symbols represent the mean concentration ± SD from 3 animals.

To assess the *in vivo* half-life of P22339, an *in viv*o PK study was conducted in rats following an IP injection of P22339. P22339 in rat serum was captured by ELISA plate coated with anti-IL-15 antibody. Anti-human IgG Fc antibody was used to detect the concentration curve. The measured *in vivo* half-life of P22339 in rat was about 13.7 hours (Fig. 6a and Supplementary Table S4). This is considerably longer than the reported <1 hour half-life of IL-15 dosed at 1 mg/kg in mice^7^.

#### PK and PD in monkey

9 healthy cynomolgus monkeys were divided into three groups and each group received a single intravenous (IV) injection at dose of 5 µg/kg, 15 µg/kg and 45 µg/kg P22339 respectively. PK curves and results were summarized in Fig. 6b and Supplementary Table S5. The half-lives of P22339 in the cynomolgus monkey were increased with doses, t_1/2_ = 2.89 hours (5 µg/kg), t_1/2_ = 5.36 hours (15 µg/kg), t_1/2_ = 8.26 hours (45 µg/kg), which showed comparable PK profiles to Ref1 molecule^30^, in particular, with very similar half-life around 5 hours at the same 15 µg/kg dose level.

**Figure 7:**
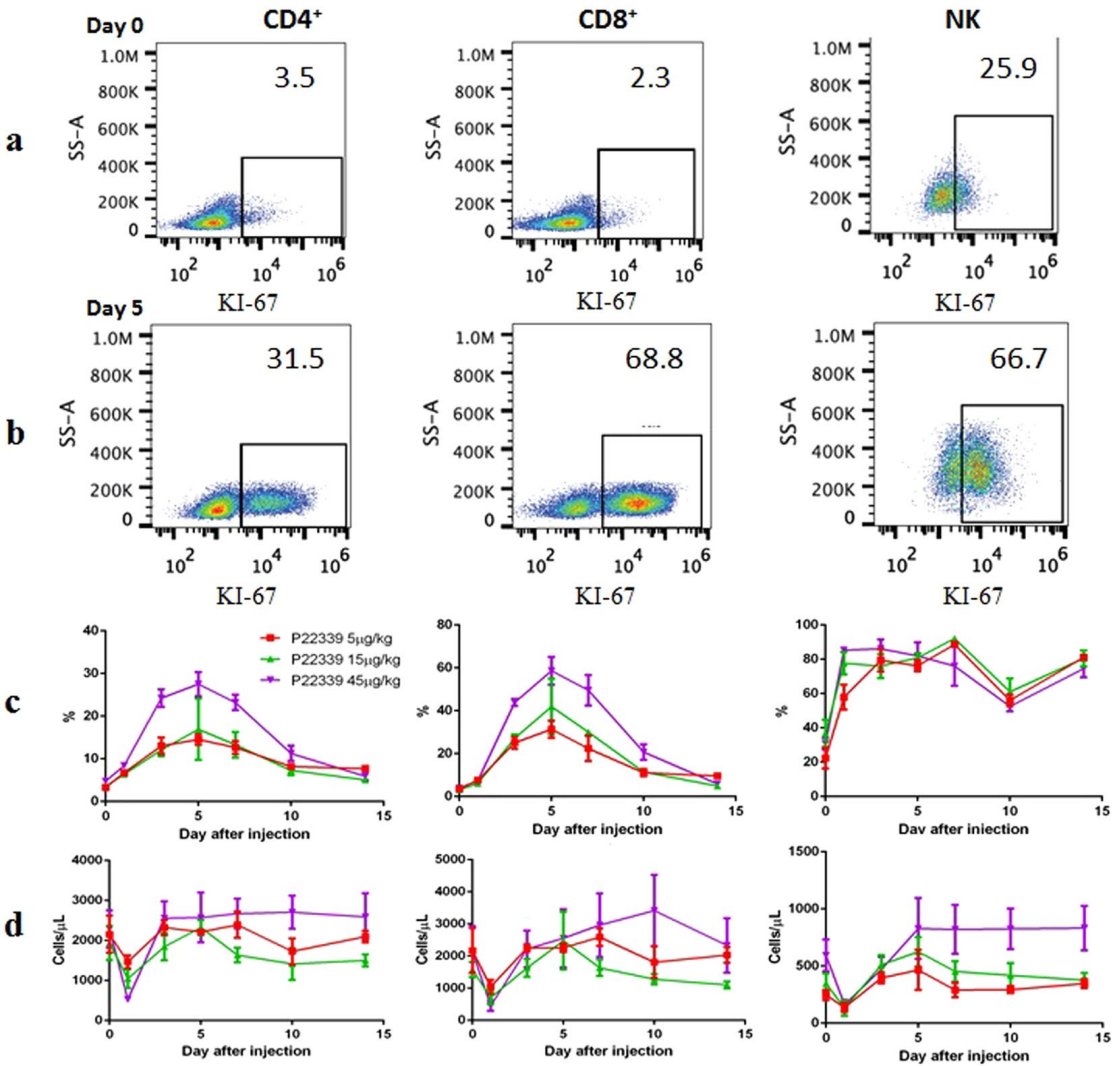
The administration of P22339 increases the percentage of immune cell proliferation. A group of cynomolgus monkeys (n = 9) were randomized into 3 groups, receiving a single IV injection of P22339 at different doses as 5 μg/kg, 15 μg/kg and 45 μg/kg. Blood were collected at different time points after the IL-15 injection at 0 d, 1 d, 3 d, 5 d, 7 d, 10 d, and 14 d, for PD analysis. Cells were stained with antibodies to CD3, CD4, CD8, CD45, CD56 and KI-67, gating on CD3^−^ and CD56^+^ for NK cells, and CD3^+^, CD4^+^/CD8^+^ for T cells. KI-67 is used as the proliferation markers for NK and T cells. (**a**) and (**b**) Representation of newly generated NK cells (right), CD8^+^ (middle), CD4^+^ (left) T cells are shown for one individual monkey administered 45 μg/kg of P22339 on Day 0 (**a**) and Day 5 (**b**). Numbers outside black lines indicate percentage of cells inside each gate. (**c**) Cell proliferation was determined as percentage of KI-67 positive cells. The symbols represent the mean ± range. (**d**) Absolute cell counts for CD4^+^ and CD8^+^ T cells and NK cells. Data are mean ± SEM, n = 3 per group.

The same groups of monkeys were also used to assess the effects of P22339 on T and NK cell subpopulations. Blood were collected at different time points at day 0, day 1, day 3, day 5, day 7, day 10, and day 14 after a single IV injection at the three dose levels mentioned above. Cells were stained with antibodies to CD3, CD4, CD8, CD45, CD56 and KI-67, gating on CD3^−^ and CD56^+^ for NK cells, and CD3^+^, CD4^+^/CD8^+^ for T cells. KI-67 was used as the proliferation markers for NK and T cells. Figure 7a,b showed the flow cytometry data for the newly generated lymphocytes and NK cells of one individual monkey administered 45 μg/kg at day 0 and day 5 respectively. Over a course of 5 days, the cell count increased 9.0, 29.9 and 2.6 folds for CD4^+^, CD8^+^ and NK cells respectively. The increased level of the marker KI-67 in blood lymphocyte and NK cells indicated that P22339-mediated effects were due to increased cell proliferation rather than merely redistribution. In comparison to the corresponding day 0 count, at all three doses tested, P22339 significantly stimulated the proliferation of T and NK cells *in vivo* indicated by the ratio of cell count at the measurement days (Fig. 7c) and by the absolute cell count (Fig. 7d). The treatments resulted in an increase in serum CD4^+^, CD8^+^ T cell and NK cell counts over two weeks period. The increase was over 80% in NK cell proliferation as soon as 1 day after dosing. For both CD4^+^ and CD8^+^ T cells, the proliferation reached up to 28% and 60% respectively 5 days after dosing. In general, the immune cell proliferations were increased in a dose proportional manner.

## Discussion

The goal of this study was to generate an IL-15 based therapy with improved potency, prolonged *in vivo* half-life, and robust developability. To make a more potent molecule, we utilized the activity enhancement effect of the trans-presentation mechanism of IL-15 and its receptor α. Fc fusion was a common way to improve the half-life of a molecule, which was also applied to IL-15 based molecule with success^30^. To further improve the developability of the molecule, an inter-molecular disulfide bond was introduced between IL-15 and its receptor α. We first examined the binding interface of the two molecules and identified candidate pairs for mutation. Although multiple pairs were identified initially, we would like to further evaluate them so that experiments could be done in a more prioritized manner. After closely examining the complex structure, we found that there were four intra-molecular disulfide bonds, two in IL-15 and two in receptor α respectively. So we classified the possibilities in two categories according to if any amino acid was near the existing intra-molecular disulfide bonds, which resulted in yes for 5 pairs and no for 4 pairs. To further narrow down the selection, we analyzed all of the pairs based on the two criteria 1) avoid the residues near the existing intra-molecular disulfide bond, 2) be energetically stable and introduce minimum change to the conformation.

Based on criteria 1, from the crystal structure of IL-15 and receptor α complex, it could be seen that in the IL-15 structure, intra-molecular disulfide bonds were formed between C35 and C85, and between C42 and C88, respectively. Therefore, it was rational to exclude E87 and E89 of the IL-15 due to their proximity to C88. Since P67 and K34 was the corresponding pairing residues in the receptor α, they were excluded as well. In addition, A37 residue of the receptor α was near the C88 in IL-15, which should be avoided to not interfere with C88. In the structure of receptor α, C29 and C63, as well as C3 and C45 formed disulfide bonds respectively. No candidate residues were found near them.

Based on criteria 2, the locations of those candidate residues in the crystal complex were analyzed. On the one hand, L45, Q48, V49, L52 and E53 were all located in an α helix in the IL-15 structure. In addition, L45, Q48 and V49 were all in the middle of that helix. If these residues were mutated to Cysteine, the torsions of the side chains caused by the formation of the disulfide bond could negatively affect the local secondary conformation, and then destabilize the whole protein structure. Therefore, L52 and E53 residues of the IL-15 were considered as preferred. On the other hand, in IL-15 Rα, L42 was located in a β sheet. A37, G38 and S40 were all located in a loop. Therefore, A37, G38 and S40 were considered as preferred. Among the pairs from the computational Disulfide Scan, L52 from IL-15 and S40 from the IL-15 Rα were considered as preferred site to form a disulfide bond.

Furthermore, introduction of disulfide bond required Cysteine mutation, which could affect the protein stability. Alanine scan was often used to assess the protein stability upon mutation. We utilized an *in silico* alanine scan method to rank order the candidate sites based on the calculated mutation energies. To further validate the results from the structure-based computational workflow, eight of the nine pairs from the Disulfide Scan were selected to be made experimentally. According to the D stability from the Disulfide Scan calculation, the least stable Pair 87–67 was excluded. The protein expression and purification results of those selected 8 pairs were in good agreement with the modeling predictions. Compared to the IL-15 and Rα Fc fusion protein without the disulfide bond (combination No. 1), six out of eight constructs with engineered disulfide bond (combination No. 4–9) showed better expression results in the western blot experiment. Among them, the four best combinations (No. 4–7) were all located far away from the existing intra-molecular disulfide bonds. Of the ones that near the existing intra-molecular disulfide bonds, No. 2 and 3 yielded no products, No. 8 and 9 showed noticeable lower yield. The results were in line with the computational predictions which further validated our structure-based disulfide design approach.

It was interesting to see that Fc-fusion on either IL-15 or Rα made a noticeable difference in terms of the production of the desired molecule (see Supplementary Fig. S2b). Early attempts also showed that fusion of IL-15 onto Fc was prone to aggregation and resulted in low yield (unpublished data).

With the trans-presentation mechanism built in, P22339 was fully capable of modulating immune responses via the IL-15R β γ complex. It showed significantly improved potency *in vitro* and *in vivo* compared to IL-15, and comparable activity to Ref1. In a B16F10 melanoma model, P22339 demonstrated antitumor immunity with dose dependent stimulation effect on T and NK cells. Even at the lower dose 5 μg/kg, the result was significantly better than IL-15 administered at the equal molar concentration. In comparison to less than one hour half-life in mice at 1 mg/kg for IL-15^7^, the Fc fusion molecule P22339 led to significantly improved t_1/2_ in rat, 13.7 hours at 188 μg/kg. Monkey PK study demonstrated that P22339 had dose proportionally increased half-life up to 8 hours at 45 µg/kg dose and exposure up to 14 µg/ml*hr. In the same *in vivo* study, we also observed the enhanced proliferation of the immune cell subpopulations in terms of the serum CD8^+^, CD4^+^ T cells and NK cells in the dose dependent manner. The disulfide bond between IL-15 and Rα sushi domain designed by *in silico* modeling further improved molecular stability. P22339 could be produced by CHO cell line with high yield and showed robust developability. Our study demonstrated that structure-based *in silico* design resulted in a potent, stable molecule with prolonged half-life and desired PD effect *in vivo*. Immunotherapy has become a clinically validated treatment option for many cancers. With the improved druggabilities, P22339 is ready to be evaluated further in cancer patients.

## Methods

### Transient expression and quantification

IL-15/IL-15Rα protein was transiently transfected and expressed by using FreeStyle 293 cells (GIBCO, Cat#R79007). FreeStyle 293 cells were cultured in Freestyle 293 expression medium (GIBCO, Cat#12338018), supplemented with Ultra Low IgG Fetal Bovine Serum (ultra low immunoglobulins FBS, GIBCO, Cat # 16250078) at a final concentration of 1%. IL-15/IL-15Rα expression plasmids and transfection reagent PEI (Polysciences, Cat#239662) were prepared, the two plasmids of IL-15 and IL-15Rα were co-transfected at a ratio ranging from 1:1 to 9:1, total amount of plasmids was 100 μg/100 ml cells, the ratio of plasmid to PEI was 1:2 by mass. Cell density on the day of transfection was 1 × 10^6^/ml. 1 L of FreeStyle 293 cells were prepared to be transfected. 50 ml of Opti-MEM (GIBCO, Cat # 11058021) medium was mixed with the plasmid, kept still for 5 min and filtered. Another 50 ml of Opti-MEM medium was mixed with PEI, kept still for 5 min and filtered. The plasmid was mixed with PEI and kept still for 15 min. The mixture of plasmid and PEI was slowly added to the cells and cultured in shaking incubator at 130 rpm at 37 °C, 8% CO_2_. 5 days later, the supernatant was collected by centrifugation for protein purification.

Ref1 molecule was prepared as previously reported^23^.

### *In vitro* Mo7e cell assay for cell proliferation

Mo7e (human megakaryocyte leukemia cell line) was purchased from Peking Union Medical College. IL-15 was purchased from Novoprotein (Cat No. C016). Ref1 was obtained from in-house preparation. Cell Counting Kit-8 (CCK-8) was purchased from WST (Cat No. EX660), and GM-CSF from NOVOProtein (Cat No. CC79).

Mo7e was cultured in modified RPMI-1640 medium (containing 2.05 mM L-glutamine, 10% FBS and 15 ng/ml GM-CSF) in the incubator at 37 °C (5% CO_2_). Mo7e cells in good condition were centrifuged at room temperature, 150 × g for 5 min. The supernatant was discarded. The cell pellet was washed with GM-CSF-free medium twice and then counted. Cell was plated in 96-well plate with a cell number of 2 × 10^4^/well and concentration adjusted to a volume of 90 μl (GM-CSF-free), and kept in the cell incubator for culture. IL-15 and its analogs were 4-times diluted with PBS, 10 μl/well was added to the cell culture system after 2 hours incubation of cells in 96-well plates. Each concentration was repeated in triplicate, blank wells (added with only PBS) were used as control. Cell plates were cultured in the incubator for 3 days. All test wells were added with 10 μl of CCK-8, and incubated in the incubator for 3 hours. Absorbance at 450 nm (OD450) was detected.

### *In vivo* study in mouse B16F10 melanoma model for Lung metastasis inhibition

All animal experimentations described in this study were conducted in accordance with the Guiding Principles in the Care and Use of Animals by the American Physiological Society. All animal experimental protocols were approved by the Institutional Animal Care and Use Committee of the Shanghai Hengrui Pharmaceutical Co., Ltd. All animals used for *in vivo* studies were treated in accordance with Institutional Guide for the Care and Use of Laboratory Animals.

32 of C57BL/6 mice (SPF, Shanghai Super B&K Laboratory Animal Corp. Ltd.) were divided into 4 groups, each group of 8 mice. 1.5 × 10^5^ of B16F10 cells were intravenously injected to mouse via tail-vein (Cell Resource Center, Shanghai Institutes for Biological Sciences, Chinese Academy of Sciences, TCM36). PBS, 2 μg of IL-15 and 5 μg or 15 μg of P22339 was intra-peritoneally injected into mouse on day 1. Weighing once every 2–3 days, one mouse from each group was killed on day 14, and the lung metastasis was observed. All mice were sacrificed on day 16. Lungs of all mice were removed and weighed, observed the black lung lumps and photographed, and then the lung was fixed in formaldehyde and counted for the number of black lumps.

### *In vivo* study in mouse B16F10 melanoma model for tumor inhibition

All animal experimentations described in this study were conducted in accordance with the Guiding Principles in the Care and Use of Animals by the American Physiological Society. All animal experimental protocols were approved by the Institutional Animal Care and Use Committee of the Shanghai Hengrui Pharmaceutical Co., Ltd. All animals used for *in vivo* studies were treated in accordance with Institutional Guide for the Care and Use of Laboratory Animals.

Mice were adapted to the laboratory environment for 5 days. C57 BL/6 mice (SPF, Shanghai Xi Puer Bei Kai Experimental Animal Co., Ltd.) were inoculated subcutaneously in the right rib with B16F10 tumor cells (5 × 10^6^/mouse). Tumor grew for 7 days. When the volume of tumor reached 160 ± 40 mm^3^, animals were randomly divided (d0) into 4 groups, each group with 7 mice. Mice were treated with PBS, IL-15 (2 μg), P22339 (5 μg), P22339 (15 μg), Ref1 (5 μg), Ref1 (5 μg) respectively, via IP injection twice on day 1 and day 5. Mice were measured for tumor volume and body weight every 2 days, and data was recorded.

### *In vivo* study in mouse B16F10 melanoma model for antitumor immunity

All animal experimentations described in this study were conducted in accordance with the Guiding Principles in the Care and Use of Animals by the American Physiological Society. All animal experimental protocols were approved by the Institutional Animal Care and Use Committee of the Shanghai Hengrui Pharmaceutical Co., Ltd. All animals used for *in vivo* studies were treated in accordance with Institutional Guide for the Care and Use of Laboratory Animals.

Murine B16F10 melanoma cells were maintained in RPMI 1640 supplemented with penicillin (100 IU/ml), streptomycin (100 μg/ml), and 10% FBS. Female C57BL/6 mice were inoculated subcutaneously at the right flank with B16F10 cells (5 × 10^5^) in 0.1 ml of 1640 medium for tumor development. The treatments were started on day 9 after tumor inoculation when the average tumor size reached approximately 300 mm^3^. Each group consisted of 4 tumor-bearing mice. Treatments were done at day 1 once by IP injections of PBS, 2 μg IL15, 5 μg P22339 and 15 μg P22339. 4 days after the treatment, the whole blood was collected in micro-anticoagulant tube. PBMCs were isolated with Histopaque®-1083 (Sigma, Cat No. 10831), and labeled with APC Rat Anti-Mouse CD45 (BD, Cat No. 559864), PE Hamster Anti-Mouse CD3ε (BD, Cat No. 553063) and FITC Rat Anti-Mouse CD49b (BD, Cat No. 553857). The percentage of T and NK cells in APC positive cells were detected with BD FACSVerseTM flow cytometer (BD).

### Statistics for *in vivo* tumor model results

Excel statistical software: mean value was calculated as avg. SD was calculated as STDEV. SEM was calculated as STDEV/SQRT. P value between different groups was calculated using t-test.

$$\begin{array}{c}{\rm{Tumor}}\,{\rm{volume}}\,({\rm{V}})\,{\rm{was}}\,{\rm{calculated}}\,\text{as}:\,{\rm{V}}=1/2\times {{\rm{L}}}_{{\rm{length}}}\times {{{\rm{L}}}_{{\rm{short}}}}^{2}\\ {\rm{Relative}}\,{\rm{volume}}\,({\rm{RTV}})={{\rm{V}}}_{{\rm{T}}}/{{\rm{V}}}_{0}\\ {\rm{Tumor}}\,{\rm{Inhibition}}\,{\rm{Rate}}\,( \% )=({{\rm{C}}}_{{\rm{RTV}}}-{{\rm{T}}}_{{\rm{RTV}}})/{{\rm{C}}}_{{\rm{RTV}}}( \% )\end{array}$$

V_0_ and V_T_ represented the tumor volume at the beginning of the experiment and at the end of the experiment, respectively. C_RTV_ and T_RTV_ represented blank control group (PBS) and relative tumor volume in the test group at the end of the experiment, respectively.

### *In vivo* pharmacokinetics study in rats

All animal experimentations described in this study were conducted in accordance with the Guiding Principles in the Care and Use of Animals by the American Physiological Society. All animal experimental protocols were approved by the Institutional Animal Care and Use Committee of the Shanghai Hengrui Pharmaceutical Co., Ltd. All animals used for *in vivo* studies were treated in accordance with Institutional Guide for the Care and Use of Laboratory Animals.

The SD rats (n = 2, provided by Sipur-Bikai Experimental Animal Co., Ltd.) were administered via IP injection with a dose of 188 μg/kg and a volume of 5 ml after fasting overnight. 0.2 ml of blood samples were taken after the administration and at 0.5 h, 1 h, 2 h, 4 h, 6 h, 8 h, 11 h, 24 h, 48 h, 72 h and 96 h. The blood samples were collected and kept in tube for 30 min at 4 °C, then centrifuged at 3500 rpm for 10 min. The serum was isolated and stored at −80 °C. The rats were fed for 2 h after dosing. All PK and TK parameters were calculated using a non-compartmental model with Phoenix WinNonlin software (5.2) (Pharsight Corporation, USA).

### Monkey PK and PD studies of P22339

All animal experimentations described in this study were conducted in accordance with the Guiding Principles in the Care and Use of Animals by the American Physiological Society. The procedures that applied on animals in this protocol were reviewed and approved by PharmaLegacy Laboratories IACUC. All animals used for *in vivo* studies were treated in accordance with Institutional Guide for the Care and Use of Laboratory Animals.

A total of 9 naïve cynomolgus monkeys (Male, 3–6 years old, 2.5–5.5 kg) were randomized into 3 groups, receiving a single IV injection of P22339 at different doses as 5 μg/kg, 15 μg/kg and 45 μg/kg. Monkeys received the specified treatment by slow push intravenous injection for 15 minutes. The study was performed to evaluate the effect of P22339 on proliferation of T cell subsets and NK cells in non-human primate. Animals were observed daily for signs of illness and general reaction to treatments. All exceptions to normal healthy appearance and behavior were recorded and detailed in standard PharmaLegacy Laboratories clinical observations forms. Blood were collected at different time points for PK or PD analysis. Such as bleeding at 0 h, 0.5 h, 1 h, 2 h, 4 h, 8 h, 24 h, 2 d, 3 d, 4 d, 5 d, 6 d, 7 d, 10 d, 14 d, 17 d and 21 d for PK samples and bleeding at 0 d, 1 d, 3 d, 5 d, 7 d, 10 d, and 14 d, after IL-15 injection for PD analysis. For all time points above, approximately 1–2 ml whole blood were collected from the cephalic or saphenous vein by using vacuum EDTA-2K tube. Gently invert the tube for several times immediately after drawing blood and then place the tube onto wet ice. For plasma preparation, it was obtained within 2 hours of blood collection by centrifugation at 2000 × g and 4 °C for 10 minutes. The antibodies specific to different immune cells marker as CD3, CD4, CD8, CD56, Ki67 *et al*. were purchased from BD science.

### Structure-based inter-molecular disulfide bond design

Disulfide Scan in MOE (Molecular Operating Environment, Chemical Computing Group Inc., Montreal, H3A 2R7 Canada, http://www.chemcomp.com, 2012) was used to identify the possible mutation pair on the interface between IL-15 and IL-15 Rα. For any given pair of residues, if their β carbons are within 5 Å of each other they were considered to be able to form disulfide bond with Cysteine mutations. The Default setting was used, Amber10:EHT as the force field and R-field as the solvation model.

### *In silico* Alanine Scanning

The effect of mutations on the protein stability was calculated using the Calculate Mutation Energy (Stability) protocol in Discovery Studio 3.5 (Discovery Studio Modeling Environment, Accelrys Inc., San Diego, CA, http://www.accelrys.com, 2012). Each selected amino acid was mutated to alanine independently. Temperature Dependent Calculations and pH Dependent Electrostatics were set to False. Preliminary minimization was turned on with CHARMm Polar H force field and default values for other parameters.

### Data availability

The data that support the findings of this study are available from the corresponding author upon reasonable request.

## Electronic supplementary material

Supplementary information
