# Supplementary material for: Discovery of a novel IL-15 based protein with improved developability and efficacy for cancer immunotherapy
Source: Sci Rep. 2018 May 16;8:7675. doi: 10.1038/s41598-018-25987-4 (PMC5955975; doi:10.1038/s41598-018-25987-4)
Supplement: Supplementary file 1 — Supplementary information [file 41598_2018_25987_MOESM1_ESM.docx]

**Supplementary Information**

**Discovery of a novel IL-15-based protein with improved developability and efficacy for cancer immunotherapy**

Qiyue Hu, Xin Ye, Xiangdong Qu, Dongbing Cui, Lei Zhang, Zhibin Xu, Hong Wan, Lianshan Zhang, Weikang Tao

Shanghai Hengrui Pharmaceutical Co. Ltd. 279 Wenjing Road. Shanghai, China 200245

**[1] Supplementary material and methods**

(1) **Affinity chromatography for IL-15 fusion protein; (2) Size exclusion chromatography for IL-15 fusion protein.**

**[2] Supplementary Figures and Tables**

**Supplementary Figure S1** : The crystal structure (PDB: 2Z3Q ) of IL-15 in complex with receptor α, β, γ

**Supplementary Figure S2** : Western analysis of the co-expression combinations of different mutations

**Supplementary Figure S3** : SEC-HPLC analysis of P22339

**Supplementary Figure S4** : Gel electrophoresis profiles of P22339.

**Supplementary Figure S5**: The proliferation of the immune cell subpopulations in the B16F10 melanoma model.

**Supplementary Table S1** : Residues at the contacting interface of IL-15 and receptor α complex

**Supplementary Table S2**: Co-expression combinations of different mutations

**Supplementary Table S3**: Tumor inhibition result using the B16F10 melanoma model in C57 mice

**Supplementary Table S4**: Pharmacokinetics parameters of P22339 in Rats

**Supplementary Table S5**: Pharmacokinetics parameters of P22339 in Monkeys

**[1] Supplementary material and methods**

**(1) Affinity chromatography for IL-15 fusion protein**

Supernatant was collected from cell culture after high speed centrifugation and subjected to affinity chromatography by using Protein A column from GE. The equilibration buffer used in chromatography was 1×PBS (pH7.4), after cell supernatant was loaded and bound, washing with PBS until UV returned to baseline, and then eluting the target protein with elution buffer (acidity, pH3.0). The pH was adjusted to neutral with Tris, and the target protein was stored.

**(2) Size exclusion chromatography for IL-15 fusion protein**

To obtain the desired product with high purity, the protein obtained during the affinity chromatography was concentrated by ultra-filtration and loaded for size exclusion chromatography by using GE Superdex200 gel to remove possible polymer and other components. Purity of the obtained protein was detected by SDS-PAGE and SEC-HPLC. Protein concentration was determined by UV spectrophotometry.

**Supplementary Figure S1**. The crystal structure (PDB: 4GS7) of IL-15 in complex with receptor α, β, γ


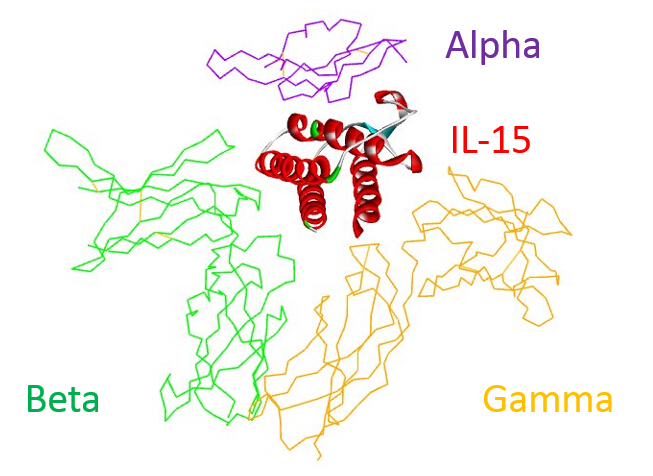


**Supplementary Figure S2**. Western analysis of the co-expression combinations of different mutations. PageRuler Prestained Protein Ladder from ThermoFisher （Product#26616）were shown on the left as the marker.


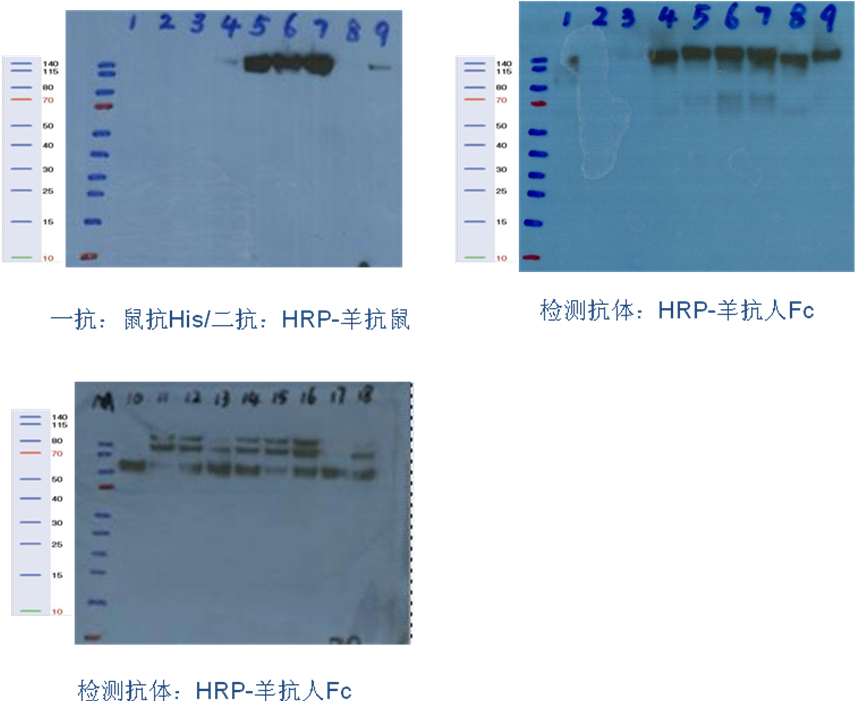

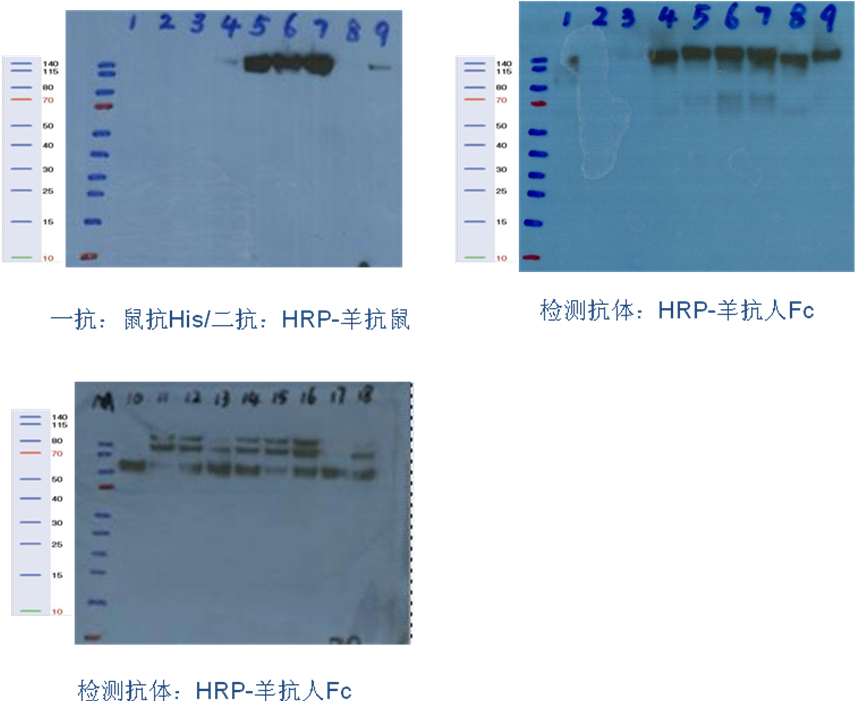


1. Co-expression combination 1-9 b. Co-expression combination 10-18

**Supplementary Figure S3**. SEC-HPLC analysis of P22339

**Supplementary Figure S4**. Gel electrophoresis profiles of P22339. a) Non-reduced and reduced SDS-PAGE of Glycosylated P22339, Lane1 Protein Marker, Lane2 Non-reduced P22339, a clear single band was found, Lane3 Reduced P22339, the lager band was IL-15 Rα - sushi (73aa) - Fc part, the three small bands were glycosylated and non-glycosylated IL-15. b) Reduced SDS-PAGE of De-Glycosylated P22339 by PNGase F, Lane4 Protein Marker, Lane5 reduced De-Glycosylated P22339, only one small band was found. Lane a and b were from two separate experiments. Both used the same PageRuler Prestained Protein Ladder from ThermoFisher (Product#26616）as the marker shown on the left.


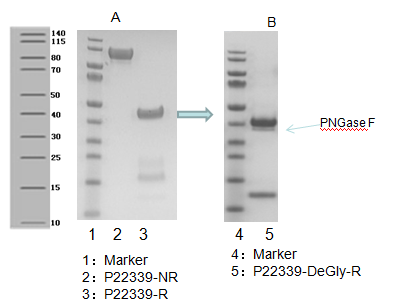


a

b

**Supplementary Figure S5**. The proliferation of the immune cell subpopulations in the B16F10 melanoma model. 16 tumor-bearing mice were randomized into 4 groups, receiving a single IV injection of PBS, IL-15, P22339 at 5 μg/kg and 15 μg/kg respectively. Whole blood were collected and PBMC were isolated on day 4 for PD analysis. Cells were stained with antibodies to CD3, CD45, CD49b, gating on CD3^+^ and CD45^+^ for T cells, and CD49b^+^, CD45^+^ for NK cells. a) FACS plots of newly generated T cells for each treatment group. b) FACS plots of newly generated NK cells for each treatment group. Numbers in each corner of the quadrant indicate percentage of cells inside each gate. The double positive cells are located in the upper right corner of each plot.

**Supplementary Table S1**. Residues at the contacting interface of IL-15 and receptor α complex

| Molecule | Residues at the contacting interface |
| --- | --- |
| IL-15 | H20, I21, D22, A23, T24, L25, Y26, C42, L45, E46, Q48, V49, L52, E53, S54, G55, E87, C88, E89, E90, E93, K94 |
| receptor α | R24, R26, K34, R35, K36, A37, G38, T39, S40, S41, L42, E44, S60, I64, R65, D66, P67, A68, V70, H71, Q72 |

**Supplementary Table S2**. Co-expression combinations of different mutations. His labels of the co-expression products for combinations 1-9 could be detected with anti-mouse His (primary antibody, abcam, ab14923) and goat anti-mouse HRP (secondary antibody, Jackson, 115-035-062). Fc part of the co-expression products for combinations 1-18 were detected with goat anti-human Fc-HRP (Jackson, 109-035-098).

| Co-expression combination NO. | Clone | Co-expression combination NO. | Clone |
| --- | --- | --- | --- |
| 1 | IL-15-his | 10 | IL-15-linker-Fc |
|  | IL-15 Rα-linker-Fc |  | IL-15 Rα |
| 2 | IL-15(L45C)-his | 11 | IL-15(L45C)-linker-Fc |
|  | IL-15 Rα(A37C)-linker-Fc |  | IL-15 Rα(A37C) |
| 3 | IL-15(L45C)-his | 12 | IL-15(L45C)-linker-Fc |
|  | IL-15 Rα(G38C)-linker-Fc |  | IL-15 Rα(G38C) |
| 4 | IL-15(Q48C)-his | 13 | IL-15(Q48C)-linker-Fc |
|  | IL-15 Rα(G38C)-linker-Fc |  | IL-15 Rα(G38C) |
| 5 | IL-15(V49C)-his | 14 | IL-15(V49C)-linker-Fc |
|  | IL-15 Rα(S40C)-linker-Fc |  | IL-15 Rα(S40C) |
| 6 | IL-15(L52C)-his | 15 | IL-15(L52C)-linker-Fc |
|  | IL-15 Rα(S40C)-linker-Fc |  | IL-15 Rα(S40C) |
| 7 | IL-15(E53C)-his | 16 | IL-15(E53C)-linker-Fc |
|  | IL-15 Rα(L42C)-linker-Fc |  | IL-15 Rα(L42C) |
| 8 | IL-15(C88)-his | 17 | IL-15(C88)-linker-Fc |
|  | IL-15 Rα(A37C)-linker-Fc |  | IL-15 Rα(A37C) |
| 9 | IL-15(E89C)-his | 18 | IL-15(E89C)-linker-Fc |
|  | IL-15 Rα(K34C)-linker-Fc |  | IL-15 Rα(K34C) |

**Supplementary Table S3**. Tumor inhibition result using the B16F10 melanoma model in C57 BL/6 mice

| Group | Dosing | Route | Ave. Tumor Vol.  （mm3） | | Ave. Tumor Vol.  （mm3） | | Relative Tumor Size | | **%Tumor Inh@D28** | #Animal |
| --- | --- | --- | --- | --- | --- | --- | --- | --- | --- | --- |
|  |  |  | D1 | SEM | D9 | SEM | D28 | SEM |  |  |
| PBS | d1/5 | i.p. | 162.39 | 16.32 | 2703.46 | 393.15 | 16.32 | 2.18 |  | 6 |
| IL-15 (2μg) | d1/5 | i.p. | 161.74 | 16.64 | 3219.01 | 644.69 | 18.32 | 2.99 | **-12%** | 6 |
| P22339 (5μg) | d1/5 | i.p. | 168.26 | 19.22 | 1892.20 | 315.12 | 11.47 | 1.58 | **30%** | 6 |
| P22339 (15μg) | d1/5 | i.p. | 168.30 | 19.17 | 824.38 | 170.63 | 4.48 | 0.61 | **73%**** | 5 |

**Supplementary Table S4**. Pharmacokinetics parameters of P22339 in Rats

| Parameter | Rat 1 | Rat 2 | Mean |
| --- | --- | --- | --- |
| t_max_ (h) | 6.00 | 6.00 | **6.00** |
| C_max_ (ng/ml) | 2135 | 1461 | **1798** |
| AUC _0-t_ (ng/ml*h) | 54789 | 44749 | **49769** |
| AUC _0-∞_ (ng/ml*h) | 54912 | 44915 | **49914** |
| t_1/2_ (h) | 10.3 | 17.1 | **13.7** |
| CLz/F (ml/min/kg) | 0.057 | 0.070 | **0.063** |
| Vz/F (ml/kg) | 50.8 | 103 | **77.0** |
| MRT _0-∞_ (h) | 16.2 | 17.5 | **16.9** |

**Supplementary Table S5**. Pharmacokinetics parameters of P22339 in Monkeys

| **Parameter** | **Unit** | **5 µg/kg** | **15 µg/kg** | **45 µg/kg** |
| --- | --- | --- | --- | --- |
|  |  | Mean ± SD (n=3) | Mean ± SD (n=3) | Mean ± SD (n=3) |
| **t_1/2_** | hr | 2.89 ± 0.46 | 5.36 ± 1.32 | 8.26 ± 1.82 |
| **C_max_** | ng/ml | 83.31 ± 11.11 | 289.14 ± 33.09 | 1238.21 ± 122.12 |
| **AUC_(0-t)_** | ng/ml*h | 367.19 ± 50.84 | 1518.1 ± 219.55 | 14195.89 ± 465.62 |
| **AUC_(0-inf)_** | ng/ml*h | 435.91 ± 77.23 | 2424.16 ± 697.71 | 16267.18 ± 1317.71 |
